# Tick-borne encephalitis virus infections in Germany. Seasonality and in-year patterns. A retrospective analysis from 2001-2018

**DOI:** 10.1371/journal.pone.0224044

**Published:** 2019-10-31

**Authors:** Johannes P. Borde, Klaus Kaier, Philip Hehn, Merle M. Böhmer, Teresa M. Kreusch, Gerhard Dobler

**Affiliations:** 1 Division of Infectious Diseases, Department of Medicine II, University of Freiburg Medical Center and Faculty of Medicine, Freiburg i.Br., Germany; 2 Praxis PD Dr. J. Borde & Kollegen / Gesundheitszentrum Oberkirch, Am Marktplatz, Oberkirch, Germany; 3 Institute of Medical Biometry and Statistics, Faculty of Medicine and Medical Center–University of Freiburg, Stefan-Meier-Straße, University of Freiburg, Freiburg, Germany; 4 Bavarian Health and Food Safety Authority, Department of Infectious Disease Epidemiology & Taskforce Infectiology/Airport Veterinaerstraße, Oberschleissheim, Germany; 5 Immunization Unit, Robert Koch Institute, Seestraße, Berlin, Germany; 6 Bundeswehr Institute of Microbiology, German National Reference Laboratory for TBEV, Neuherbergstraße, München, Germany; Faculty of Science, Ain Shams University (ASU), EGYPT

## Abstract

**Background:**

Little is known regarding the changing seasonality of infections with the tick-borne encephalitis virus (TBEV) and the incidence of the resulting disease over the last two decades. Seasonal patterns have to our knowledge not previously been systematically investigated and are poorly understood. We investigate emerging seasonal changes in clinical aspects like potentially increasing hospitalization during the year, variations in clinical symptoms and disease severity during the season and seasonal dynamics of fatal outcomes.

**Material and methods:**

TBEV infection became a notifiable disease in Germany in 2001. We used the national reporting dataset spanning from 2001–2018, provided by the Robert Koch-Institute (RKI). There were general epidemiological variables available, including “symptom onset”, “age” and “sex”. Furthermore, several variables documented disease severity. These included “CNS symptoms”, “myelitis”, “fatal outcome” and “hospitalization”. Potential factors influencing the occurrence of CNS symptoms, myelitis, hospitalizations and fatal outcome were analyzed using logistic regression models. Linear trends, including the “time point in year” at which TBEV infection related symptoms were detected, were tested using calendar year as a continuous covariate. In addition, seasonal trends and age and sex specific differences were exploratively tested for non-linear effects using restricted cubic splines with knot locations based on Harrell's recommended percentiles. Finally, the dynamic relationship between in-seasonal trends year of detection, sex and age was tested using interaction terms.

**Results:**

6,073 TBEV infection cases from 2001–2018 were included in our analysis. We find that from 2001–2018 TBEV infections are reported 0.69 days earlier each year (p<0.001). There was no detectable seasonal variation regarding the occurrence of fatal outcome, CNS and myelitis. However, there was a significant changing trend regarding hospitalizations over the course of the year: The risk for hospitalization increases until August, decreases again from October on.

**Conclusion:**

We present epidemiological evidence that the TBE season in Germany has shifted to start earlier over the last years, beginning approximately 12 days earlier in 2018 than it did in 2001. There are seasonal patterns regarding a higher risk of hospitalization during August.

## Introduction

Tickborne encephalitis (TBE) is endemic in Central Europe, in Eastern Europe, in parts of Northern Europe and especially in the Baltics. TBE is the most important arboviral disease in Europe and Northern Asia, with 10.000–15.000 cases each year [1]. TBE is caused by the tick-borne encephalitis virus (TBEV), which is a member of the *Flaviviridae* family [2,3]. TBEV shares many genetic features with other mosquito-borne and tickborne flaviviruses, such as Dengue virus (DENV), Zika virus (ZIKV), Yellowfever virus (YFV) and Powassan virus (POWV). This virus’s first scientific description dates back to 1938, as part of a dramatic effort to combat an epidemic of encephalitis among troops in the far east of the USSR. A vaccine was quickly developed and put into use in 1939 [4].

Five genetic subtypes of TBEV are currently acknowledged to exist, the Baikalian, the Far Eastern, the Himalayan, the Siberian, and the Western subtypes, of which the Baikalian and Himalayan are relatively recent discoveries [5]. Vaccination is the main defense against the virus, since as of now no specific antiviral treatment exists. Once it has occurred, TBEV infection can lead to severe and sometimes lasting ill health effects and even death, with a mortality rate of 1% reported for the Western type that is prevalent in Germany. This of course is linked to a considerable use of medical resources [1,6–8]. The available vaccines, Encepur^®^ by GSK and FSME Immun^®^ by Pfizer, fortunately offer very good protection and are safe, with more than 98% of patients completing the basic vaccination schedule exhibiting seroconversion [9]. However, for a variety of reasons many of those at risk in Europe are not vaccinated, with vaccination rates often far below the >85% seen in Austria, the country with the highest rate. In Germany, only 27% of the population have ever received even a single TBEV shot [10].

Several aspects of TBE epidemiology, TBE disease and vaccination remain a matter of debate. Descriptive results using the national surveillance dataset were published before by Hellenbrand et al. [11], however long-term trends have been studied with a particular focus on the geographic spread and related dynamics of human TBEV infections [11,12], in order to detect high-risk regions. We want to answer questions regarding the changing seasonality of TBE incidence over the last two decades. Seasonal patterns have to our knowledge not been systematically investigated previously and are poorly understood. We investigate emerging seasonal changes in clinical aspects like potentially increasing hospitalization during the year, variations in clinical symptoms and disease severity during the season and in-season dynamics of fatal outcomes.

## Materials and methods

### Dataset and definitions

TBEV infections became a notifiable disease in Germany in 2001. National (by the German Robert Koch Institute, RKI) and European case definition (by the European Centre of Disease Prevention and Control, ECDC) criteria have been issued. The dataset and definitions of clinical variables have been published before by Hellenbrand et al. [11].

In detail [11] these variables are characterized as following: “either non-specific symptoms or central nervous system (CNS) symptoms indicating CNS infection (meningitis, encephalitis or myelitis separately or in combination) and laboratory confirmation of either simultaneously elevated IgM and IgG TBEV-specific antibodies in serum or cerebrospinal fluid (CSF), an increase in TBEV-specific IgG antibodies in two serum samples or the detection of intrathecal antibody synthesis [13]. Until 2004, the detection of increased IgM TBEV-specific antibodies was considered sufficient for laboratory diagnosis. General epidemiological variables documented included “symptom onset”, “age” and “sex”. Furthermore, several variables documented disease severity. These included “CNS symptoms”, “myelitis”, “fatal outcome” and “hospitalization”. In detail, “CNS symptoms” defines the presence of unspecific symptoms or specific CNS symptoms. The item “myelitis” has been reported separately since 2001. There is detailed information included regarding vaccination history of the patients.[11]”

### Statistical methods

Potential factors influencing the occurrence of CNS symptoms, myelitis, hospitalizations and fatal outcome were analyzed using logistic regression models. Linear trends, including the “time point in year” at which TBEV infection related symptoms were detected, were tested using a linear regression analysis with calendar year as a the single continuous explanatory variable. For missing values regarding onset of symptoms median imputation using the reporting date was applied on an annual basis. This means that the annual mean difference between onset of symptoms and reporting date was imputed. The endpoints myelitis, CNS symptoms, hospitalization and fatal outcome were analyzed for seasonality using logistic regression models. Therefore, the “time point in year” at which TBEV infection related symptoms were detected was included as a continuous but non-linear covariate. The non-linearity was modelled using restricted cubic splines with knot locations based on Harrell's recommended percentiles [14]. Based on the results of these logistic regression models, predicted probabilities were calculated and explored graphically. Finally, the dynamic relationship between in-seasonal trends, year of detection, sex and age was tested using interaction terms. In case that the p-value of the interaction term indicated different in-seasonal trends for specific subgroups, these differences were explored graphically. All analyses were conducted using STATA 15.1 (College Station, Texas, USA) and Microsoft Excel^®^ software.

## Results

### General epidemiology

6,073 TBEV infection cases from 2001–2018 were included in our analysis, regardless of the vaccination status. Sufficient information on the vaccination status was available for 5,777 cases. 5,298 presented with a native TBEV infection and 479 with an infection despite having been vaccinated at least once. Descriptive results using this set of data were published before by Hellenbrand et al. [11]. For a general study population overview see Table 1, which has been published/submitted in parts before with a different research background and question than this manuscript.

**Table 1:** Overview study population (N = 6,073) and dataset, general epidemiology.

		mean	SD
Demographics			
	age	46.6	19.4
		**n**	**%**
	male	3857	63.5%
	female	2211	36.4%
Symptoms			
	no CNS symptoms	3122	51.4%
	CNS symptoms	2800	46.1%
	missing	151	2.5%
Hospitalization			
	hospitalized	4844	79.8%
	not hospitalized	1129	18.6%
	missing	100	1.6%
Outcome			
	survived	6022	99.2%
	fatal	25	0.4%
	missing	26	0.4%
Myelitis			
	no myelitis	5885	96.9%
	myelitis	188	3.1%
	missing	0	0.0%

### Seasonality, the long-term perspective, 2001–2018

June and July had the highest frequency of TBEV infections (S1 Fig). Analyzing the development over the years we found that TBEV infections are reported 0.69 days earlier each year (p<0.001). In addition to the date at which TBE cases were reported (notification date), the date of disease/symptom onset was available for 88% (N = 5,323) of the TBE cases. On average, disease onset was 19.07 days [95%CI 18.50–19.64] before the cases were reported. Furthermore, the onset of symptoms showed a comparable trend (0.67 days earlier each year, p<0.001) as the reporting date. The TBE season in Germany thus started approximately 12 days earlier in 2018 than it did in 2001.

### Seasonal trends and patterns, 2001–2018

**Fig 1:**
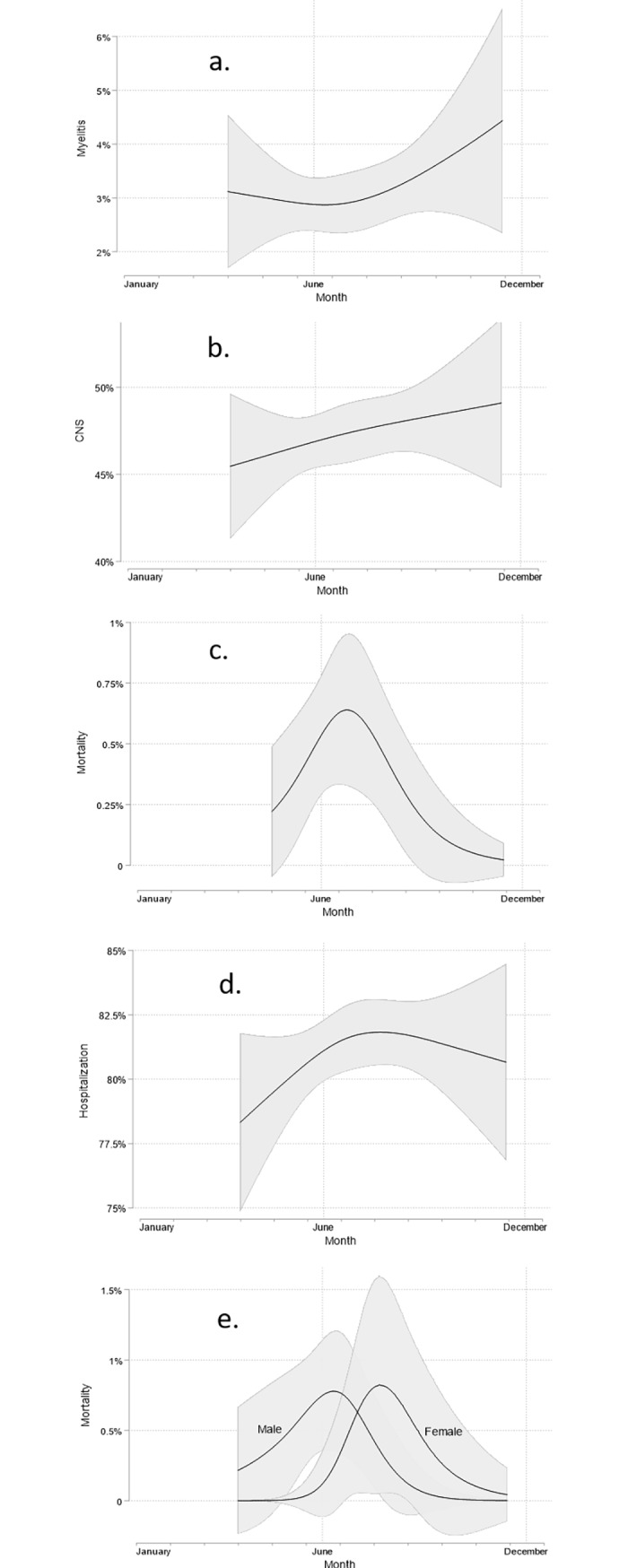
Seasonal analysis—It is shown, that there was no detectable seasonal variation regarding the variables myelitis, CNS and fatal outcome (Fig 1). However, there is a significant seasonal trend regarding hospitalization, which increases until August (d) It is shown that there was no detectable seasonal variation regarding the variable fatal outcome (c). In (e), female patients who died, had a later onset of symptoms than their male counterparts. Predicted probabilities and corresponding 95% confidence intervals (shaded area) based on the results of logistic regression models with the time point in year at which TBEV infection related symptoms were detected included as a continuous but non-linear covariate. Non-linearity was modelled using restricted cubic splines with knot locations based on Harrell's recommended percentiles [14].

We further analyzed whether there was a significant in-year seasonality in the variables fatal outcome, hospitalization, CNS and myelitis. There was no detectable seasonal variation regarding the variable myelitis, CNS and fatal outcome (Fig 1). However, there was a significant trend in hospitalizations over the course of the year (Fig 1). The risk for hospitalization increases until August, and then decreases again from October on.

### Subgroup-specific changes of in-season trends and patterns

Sex-specific differences in seasonal trends were observed for the endpoint fatal outcome only (p-value of interaction term 0.088). As shown in Fig 1 female patients, who died, had an onset of symptoms 38.98 days later (p = 0.007) than their male counterparts. Age-specific differences in seasonal trends were not observed (all p-value of interaction terms >0.1).

**Fig 2:**
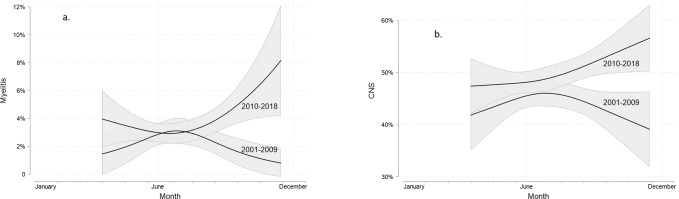
For the early years (2001–2009), an autumn decrease of CNS and myelitis was detected. For the later years (2010–2018), however, there is a steep increase in CNS and myelitis occurrence in autumn. Predicted probabilities and corresponding 95% confidence intervals (shaded area) based on the results of logistic regression models with the time point in year at which TBEV infection related symptoms were detected included as a continuous but non-linear covariate. Non-linearity was modelled using restricted cubic splines with knot locations based on Harrell's recommended percentiles [14].

As shown in Fig 2, the seasonal trend of CNS and myelitis occurrence changes substantially over the years (p-value of interaction terms: 0.007 and 0.001, respectively). For the early years (2001–2009), an autumn decrease of CNS and myelitis was detected. For the later years (2010–2018), however, there is a steep *increase* in CNS and myelitis occurrence in autumn. No such changes in trend were observed for the other variables.

## Discussion

The analyzed dataset provides evidence of a shift of the TBE season in Germany towards an earlier beginning, by approximately 12 days over the last eighteen years. Such a shift was proposed before on the basis of large dataset from the Czech Republic 1970–2008, however compared and analyzed on a coarser time scale, in 10 year blocks [15]. The link with climate change is obvious, however interactions are complex and poorly understood. TBE infections and reported TBE cases are mainly influenced by two variables, first human exposure or outdoor activity and second by tick activity itself. Both factors are linked to a plethora of interplays, which include wildlife cycles, socioeconomic factors, meteorological parameters like temperature, precipitation rate and air humidity [16,17]. Overall, the number of TBE cases seems to be more dependent on host activity than on tick activity itself [18,19]. It is speculated that climate change increases host outdoor activity, i.e. earlier outdoor and recreational activity, more than it affects vector cycles. It might be of future research interest to identify factors which predict or affect human activity–e.g. perceived temperature or beginning of the vegetation period. The earlier beginning of the TBE season should prompt health care providers and health care authorities to anticipate emerging TBE cases as early as March or early April.

The seasonal trend of an increasing hospitalization rate, peaking in August/September, is a new finding. A seasonal dynamic of the item “fatal outcome” shows in the summer months, but without reaching statistical significance. Possible explanations for the hospitalization phenomenon include virological and hospital-economic factors. Virological considerations include the seasonal occurrence of different pathogenic TBEV quasi-species, and varying TBE viral load in ticks over the season, which might cause different clinical forms. However, these hypotheses are far from being proven. In particular there is no correlation to other clinical variables like CNS and myelitis. There might also be a hospital-economic effect, resulting in a lower threshold for hospital admittance during the summer months. This period is typically associated with a decreased number of inpatients due to seasonal holidays and low season for respiratory tract infections like influenza. For the early years (2001–2009), an autumn decrease of CNS and myelitis was detected. However, in the later years (2010–2018), there was even a strong *increase* of CNS and myelitis occurrence in autumn. These findings are based on large numbers (n = 2800 for CNS, n = 188 for myelitis), but difficult to explain. From a virological point of view one might again speculate about changing seasonal pathogenicities of different TBEV quasispecies over the years, but, as mentioned above, these hypotheses are far from being proven. Another approach to explain these findings would be the increase in the number of magnetic imaging (MRI) procedures over the last ten years—especially in MRI procedures of the CNS. This might lead to a higher number of radiological myelitis diagnoses, which are not always in-line with the presence of clinical symptoms for myelitis.

There is a seasonal difference regarding the variable fatal outcome between male and female patients. Female patients, who died, had an onset of symptoms later during the year than their male counterparts. The results must be interpreted with caution in the context of the overall small number of patients with a fatal outcome (n = 25).

Our study has several limitations. In view of the used dataset, there is no scoring of disease severity as was proposed in previous literature [20], nor a source data verification of the reported cases was done. Clinical information is exclusively derived from formalized interviews, which were conducted by the public health authorities. In detail, public health physicians and public health officers contact the patient, hospital care providers and/or primary care providers to gather data. This might result in a unknown number of incorrect reported and documented variables. Furthermore, the time of the interview is not defined, therefore some cases are contacted very early in the course of the disease and other delayed.

In conclusion, we present epidemiological evidence that the TBE seasons starts earlier over the last years. There are in-seasonal patterns regarding a higher risk of hospitalization during August.

## Supporting information

S1 Fig Overall cumulative frequency of reported TBEV cases (diseases onset) over the year spanning the study period 2001–2018. Each month is subdivided into four bars. Peak frequency is detected in the months June and July.
(TIF)
