# Supplementary figures and images for: Tick-borne encephalitis virus infections in Germany. Seasonality and in-year patterns. A retrospective analysis from 2001-2018
Source: PLoS One. 2019 Oct 31;14(10):e0224044. doi: 10.1371/journal.pone.0224044 (PMC6822726; doi:10.1371/journal.pone.0224044)

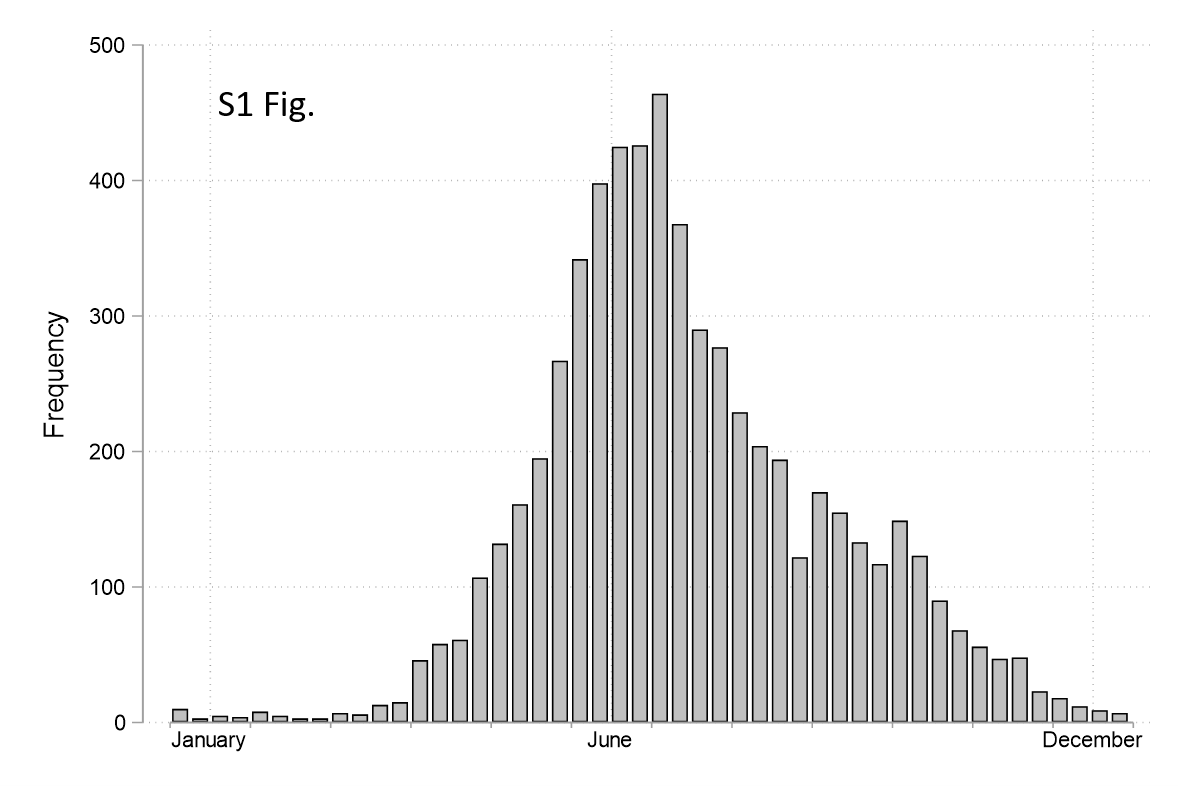

Supplement: S1 Fig — Each month is subdivided into four bars. Peak frequency is detected in the months June and July. (TIF) [file pone.0224044.s001.tif]
